# Self-assessment of health promoting Hospital’s activities in the largest heart Hospital of Northwest Iran

**DOI:** 10.1186/s12913-018-3378-1

**Published:** 2018-07-20

**Authors:** M.-H. Taghdisi, S. Poortaghi, V. Suri-J, T. Dehdari, M. Gojazadeh, M. Kheiri

**Affiliations:** 1 Health Education and Promotion Department, Faculty of Health, Iran University of Medical Sciences, Tehran, Iran; 2 School of Nursing and Midwifery, Tehran University of Medical Sciences, Tehran, Iran; 3 School of Nursing and Midwifery, Tabriz University of Medical Sciences, Tabriz, Iran; 4 Health Education and Promotion Department, Faculty of Health, Iran University of Medical Sciences, Tehran, Iran; 5 Liver and Gastrointestinal Disease Research Center, Tabriz University of Medical Sciences, Tabriz, Iran; 6 Faculty of Health, International Campus, Iran University of Medical Sciences, Second floor, school of health, Iran University of Medical Sciences, Hemmat highway, Next to Milad Tower, Tehran, Postal Code 14665-1579 Iran

**Keywords:** Health-promoting hospital, Standards, Self-assessment

## Abstract

**Background:**

Health Promoting Hospitals are among the major health promoters of the society. To acquire Health Promoting Hospital (HPH) status, a hospital must self-assess to know their inadequacies and then lay the foundation for improvements. This study has been performed with the aim of assessing readiness of the largest heart hospital of northwestern Iran regarding the HPH standards.

**Methods:**

This cross-sectional study was conducted through the participation of 270 administrative and clinical staff of the largest heart hospital of northwestern Iran. Data were gathered using *self-assessment tool* for health promoting hospitals including demographics and the HPH standards. HPH standards’ dimensions were Management policy, Patient assessment, Patient information and intervention, Promoting a healthy workplace, and Continuity and cooperation. Analysis was performed by SPSS v. 16 with a significance level of 0.05.

**Results:**

The participants included clinical (67.4%) and administrative (32.6%) staff. Among the HPH standards, the lowest score belonged to the management policy (1.44 ± 0.53) and the highest one to the patient information and intervention (1.72 ± 0.47). The average score of compliance with the HPH standards was 1.60 ± 0.40 which shows moderate progress of the hospital towards the HPH standards.

**Conclusion:**

Regarding the moderate situation of the hospital in HPH standards and the low score of the management policy, the studied hospital should enforce the standards, especially in the management policy. Also, there is a need for health promotion programs in all three levels of prevention with the participation of the staff and the patients as much as possible.

## Background

The Ottawa Charter is released by the World Health Organization (WHO) to promote health in various aspects of the societies such as schools, hospitals, colleges, workplaces, and families. It introduces health as a positive and common concept in all countries and cultures [1–5]. Health promotion (HP), as defined by the Ottawa Charter, includes health education, disease prevention, and rehabilitation [6] which are practiced by enabling the patients and their families and also the health workers to improve the physical, mental and social welfare [6, 7].

Hospitals are one of the most important environments for health promotion at all three levels of prevention [8] and the presence of patients, families and health workers makes it possible to practice HP in all three levels of the prevention [9, 10]. WHO introduced the Health Promoting Hospitals (HPH) model in 1988 with the aim of improving health and preventing diseases [1, 2]. Hospitals participate in HP by improving personnel capabilities, treatment and rehabilitation of diseases, providing facilities, and by involving workers and patients [11]. These activities are put into action via improving communication and information in decision making and also by improving the organization’s environment such as smoke-free environment [12]. HP activities in the hospitals lead to reduction in the morbidity and mortality, improved outcomes, and reduced treatment costs [13] also, these activities cause to make satisfaction of hospital’s internal and external stakeholders such as patients, staffs, relatives and other visitors, prevention of Non-Communicable Disease(NCDs) and quality of life improvement [8].

Considering the important role of the hospitals in modification and re-orientation of the health service in the community, it is necessary to have some standards for improvement of health and for assuring the quality within the hospitals [14]. Such standards were developed by the WHO European Regional Office in 2004 for HPH [15] that consist of management policy, patient assessment for health risks and needs, patient information and intervention for health, promoting a healthy workplace, and continuity of care and cooperation with other healthcare providers [11].

As search results showed, few studies have been performed in Iran on the HPH and the literature is limited [16]. Amiri et al. reported that although implementing the HPH standards is difficult in Iranian hospitals, it would lead to improvements in the hospital indicators such as percentage of bed occupancy, average length of stay, bed turnover interval, mortality & morbidity and patients’ satisfaction etc. [17, 18]. Johnson and Baum also stated that the standards require a suitable infrastructure along with well-educated human resources [19]. another study which investigated experts opinion about health promotion interventions in hospitals showed that about 63% of experts believed that these services were not offered at all in hospitals, and 37% believed that these services are considered scattered and non-organized, regarding the status of the provision of health promotion services, 87% of the experts believed that these HP activities are provided personalized and non-organized [9]. Other studies on the necessity of the HPHs indicated that moving towards the HPH is essential for controlling the disease consequences, increasing the capacity to deal with health-related problems, and changing the health behaviors of the people, all of which would result in improved level of health, welfare, and reduced financial burden [20, 21]. Previous studies have shown that organizing the healthcare services with the aim of HP according to the Ottawa Charter requires changes in strategies, professional behaviors, and organizational culture and structure [22]. Estebsari et al. stated the necessity of HP standards in the hospitals that aim to enable the patients and to improve the personnel who are exposed to physical and mental hazards [2].

Madani Heart Center (the largest heart hospital of northwestern Iran) in recent years has made great efforts to join the HPHs. However, to achieve this goal, the level of HPHs’ standards should be evaluated at this center in order to identify the barriers and facilitators of the implementation [11], and promotion plans should be made. Therefore, this study was conducted to assess the standards of HPHs in Madani Heart Center using *self-assessment tool* for health promoting hospitals [10, 11, 15, 23].

## Methods

This cross-sectional study had been conducted in the Madani Heart Center of Tabriz city, East Azarbaijan Province, Iran in the year 2016. The hospital is a single specialty Heart hospital which is the largest of its kind in the northwest of the country. The participants were administrative and clinical staff of the hospital. The sample size was calculated to be 270 [17]. The participants were selected by convenience sampling method and purpose of study was explained to the participants and informed consent was attained.Inclusion criteria were working at the hospital for at least one year, and willingness to participate in the study. Exclusion criterion included incomplete completion of the questionnaire.

The study tool was a self*-*assessment tool for health promoting hospitals [10, 11, 15, 23] that consisted of two parts: first the demographics (age, sex, education, work experience [years], post); and second the main body of the international questionnaire that included 40 items in 5 dimensions. The dimensions of the questionnaire were Management policy (9 items), Patient assessment (7 items), Patient information and intervention (6 items), Promoting a healthy workforce (10 items), and Continuity and cooperation (8 items. In total, 40 measurable items were evaluated as yes (2 points), no (no points), and to some degree (1 point) [11, 17, 24].Validity and reliability of the tool was investigated and proved by Groene (2010) in 38 hospitals of 8 member countries in the International Network of Health Promoting Hospitals and Health Services and Cronbach’s Alpha was reported to be 0.77 to 0.88 [10]. Reliability and validity of the scale was confirmed by the Ministry of Health and Medical Education [17].

A total of 270 questionnaires were distributed among the administrative and clinical staff and they invited to participate in the study, all participants were assured of the fact that their participation was voluntary in the study and all of their information would be kept confidential by not mentioning their names in the questionnaires. The gathered data were entered into the SPSS 16 software and presented by descriptive statistics. Chi-square test (for nominal variables such as staffs’ post and HPH determinants/standards.), U Mann-Whitney(in replace of t-test and compared the effect of determinants among two group of staff;administrative and clinical), and the Spearman Correlation Coefficient(for determining the relationship between an ordinal variable and a dependent quantitative one such as year of experiences with HPH determinants) were used for analysis. All statistical tests were performed with the 0.05 significance level. This research, which was a part of the Ph.D. thesis has been approved by IR.IUMS.REC 1395.9223489202 code in Ethics Committee of Research Deputy of Iran University of Medical Sciences.

## Results

Of the 270 questionnaires filled, 12 were incomplete and then excluded from the analysis. 49.23% (127) of participants were male. The average age and work experience of the participants was 40.31 ± 12.5 and 15.71 ± 6.71, respectively, and 67.4% of the staff were clinical staff. Among the demographic variables, there was a statistically significant correlation between the organizational post and the HPH (*P* < 0.001, X^2^ = 23.18). The Spearman Correlation Coefficient also showed a correlation between work experience (years) and the HPH (*P* < 0.03, *r* = 0.185). According to Table 1, there was no relationship between other demographic characteristics with HPH standards.

**Table 1:** Relationship between employee demographic characteristics and HPH standards

Demographic variables		Number (%)	Standard total scores Number (%) Yes, Partly, No	Standard total Mean ± S.D	Test	*P* value
Education level	Diploma and Associate Degree	*(4.65) 12*	1451 (14.02), 3529 (34.99), 5488 (50.84)	*0.40 ± 1.60*	٭χ^2^ = 2.33	*0.67*
	Bachelor	*(76) 196*				
	Master of Science	*(18.55) 48*				
	Ph.D.	*(0.8) 2*				
Job position	Supervisor and head nurse (middle managers)	*(7.76) 20*			*23.18 =* ^*2*^*χ*	*0.00*
	Nurse	*(57.36) 148*				
	Nurse assistance	*(6.3) 16*				
	Official	*(17.05) 44*				
	Radiologists	*(4.65) 12*				
	Senior Managers	*(6.97) 18*				
Service unit	Medical	*(67.4) 174*			*1799.500 = u ٭٭*	*0.89*
	Official	*(32.6) 84*				
Work experience [years]		*6.71 ± 15.71*			*٭٭٭r = 0.185*	*0.03*

r *** = Spearman Correlation Coefficient u ** = U Mann-Whitney *χ* ∗ *=*Chi-square

Among the five dimensions of the HPH, the lowest score belonged to the Management policy (1.44 ± 0.53) and the highest one to the dimension of Patient information and intervention (1.72 ± 0.47). The overall mean score and standard deviation of the five dimensions of HPH standards was 1.60 ± 0.40 which shows moderate compliance with them. The scores on the HPH standards are presented in Table 2.

**Table 2:** Mean and standard deviations of standards

Standard	Mean ± S.D	Confidence interval
1. Management policy (*N* = 9)	*0.53 ± 1.44*	*1.39–1.53*
2. Patient assessment (*N* = 7)	1.68 ± 0.43	*1.60–1.75*
3. Patient information & intervention (*N* = 6)	*0.47 ± 1.72*	*1.64–1.81*
4. Promoting a healthy workplace (*N* = 10)	*0.44 ± 1.53*	*1.45–1.60*
5. Continuity & cooperation (*N* = 8)	*0.44 ± 1.70*	*1.63–1.78*
Hospital total score (*N* = 40)	*0.40 ± 1.60*	*1.53–1.67*

N=Number of sub-standard items

**Fig. 1:**
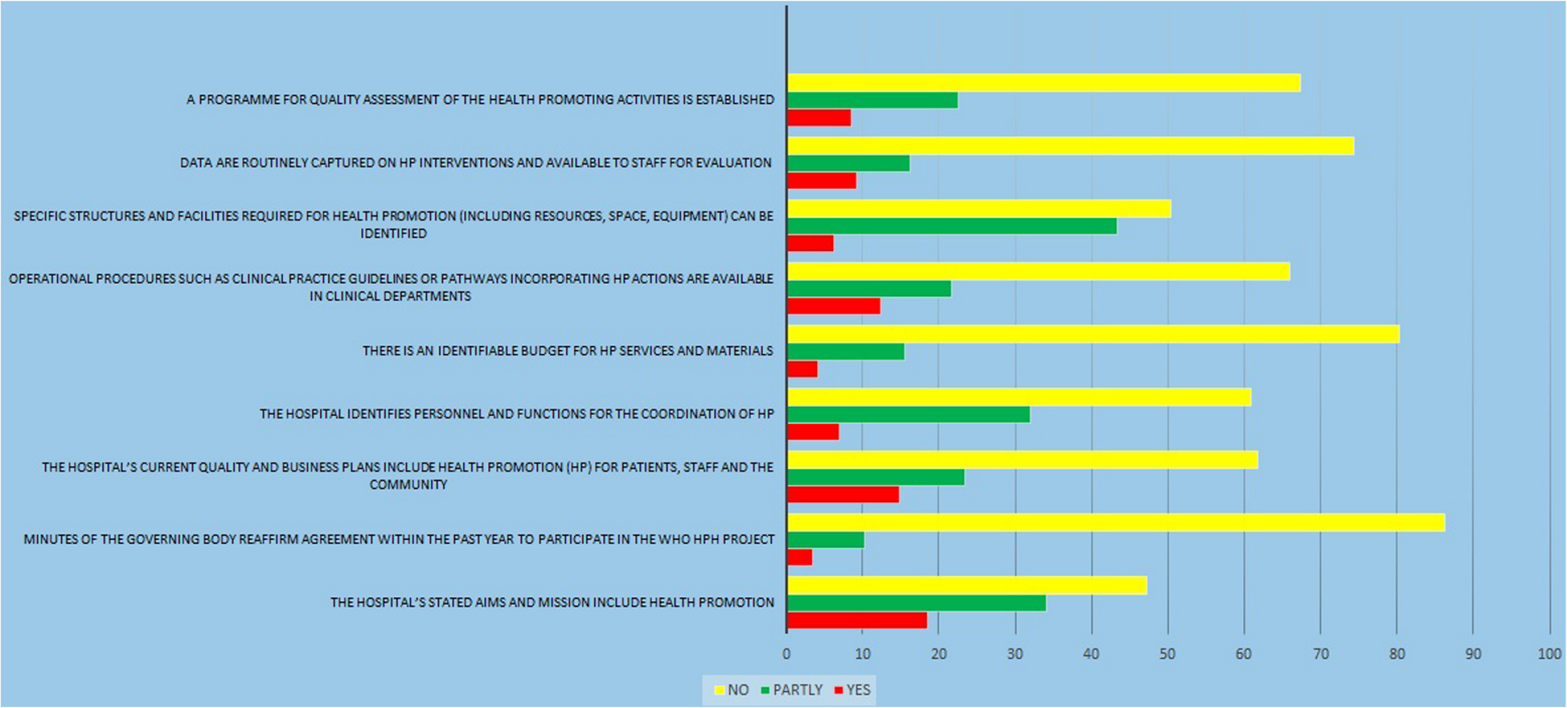
Standard 1; Management Policy

As Fig. 1 shows, the initial standards of management policy are implemented at the lowest level and have barriers such as the lack of a specific budget, program, facilities and individuals for HP, as well as there being almost no specific plans for quality assessment of the health promoting activities. On the other hand, the results indicate that the goals of the hospital in this dimension are almost as good as health promotion.

**Fig. 2:**
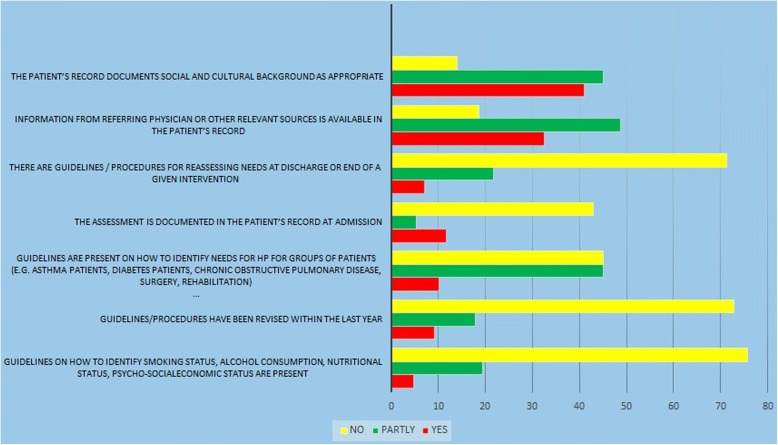
standard 2; patient assessmnet

On the second dimension (Fig. 2), which showes the standard of patient assessment based on HPH, hospital guarantees that the health promotion needs of the patients are assessed systematically and with contribution of them. The results on this dimension showed that the hospital had a low ranking in this dimension (standard). Also, there are some barriers in this dimension such as the lack of specific guidelines for identifying needs for HP for differrent groups of patients, as well as for reassessing needs at discharge, and in cases of smoking and alcohol.

**Fig. 3:**
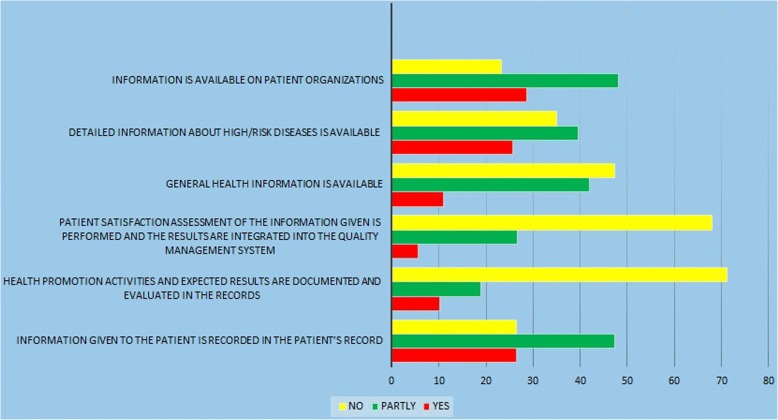
Standard 3; Patient Information and Intervention

In the third dimension (Fig. 3), the activities are focused on informing the patients about the health situation, the related factors, and the therapeutic plans. The patients’ ability is determined by active contribution in the decisions and interventions. In this dimension the activities of assessing the sufficiency of provided information to patients, and integrating the results in the quality management system had the lowest scores (5.5%). Totally the activities in this dimension were 17.82% compliant and 48.1% partially compliant with the standards of the HPH. Nevertheless, in this dimension, there were some barriers for lack of some functions in line with standard three, such as lack of information on general health, and evaluations of patient satisfaction, as well as HP activities, and the results of these activities that were recorded were not available.

**Fig. 4:**
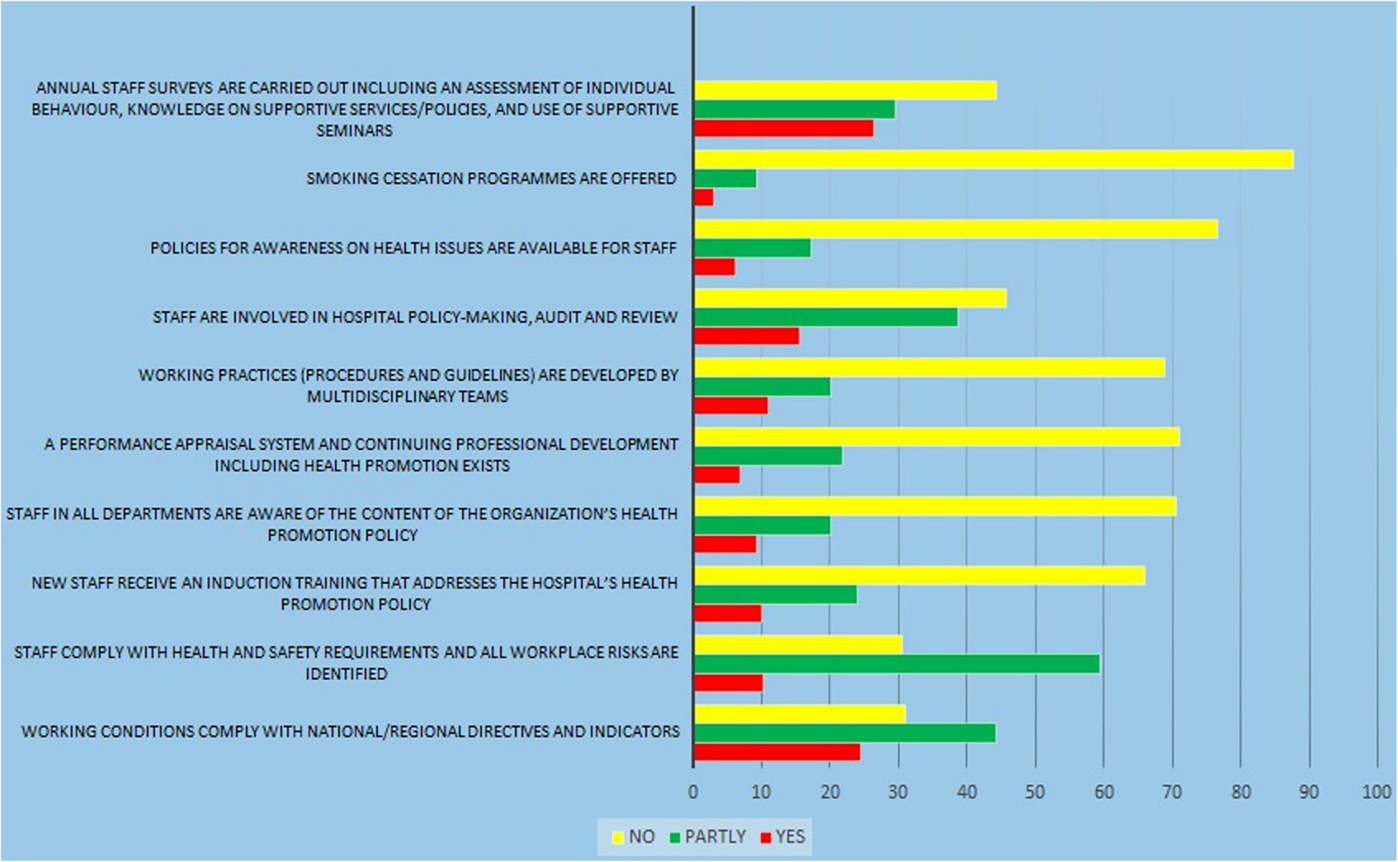
Standard 4; Promoting a Healthy Workplace

The fourth dimension (Fig. 4) which is related to make a healthy and safe environment for the visitors and the workers based on fourth standard of HPH. In this dimension the results showed that the hospitals’ HP activities for creating healthy and safe environment was (44.4%) comply with the HP national/regional guidelines and indicators. The personnel awareness about health and safety needs and identifing the environmental risks was about (59.4%). But in the aspects of quit smoking (3.1%), involvement in HP activities (15.5%), knowledge of personnel on HP activities (9.3%), and the existence of organizational policies regarding the HP was (6.3%).

**Fig. 5:**
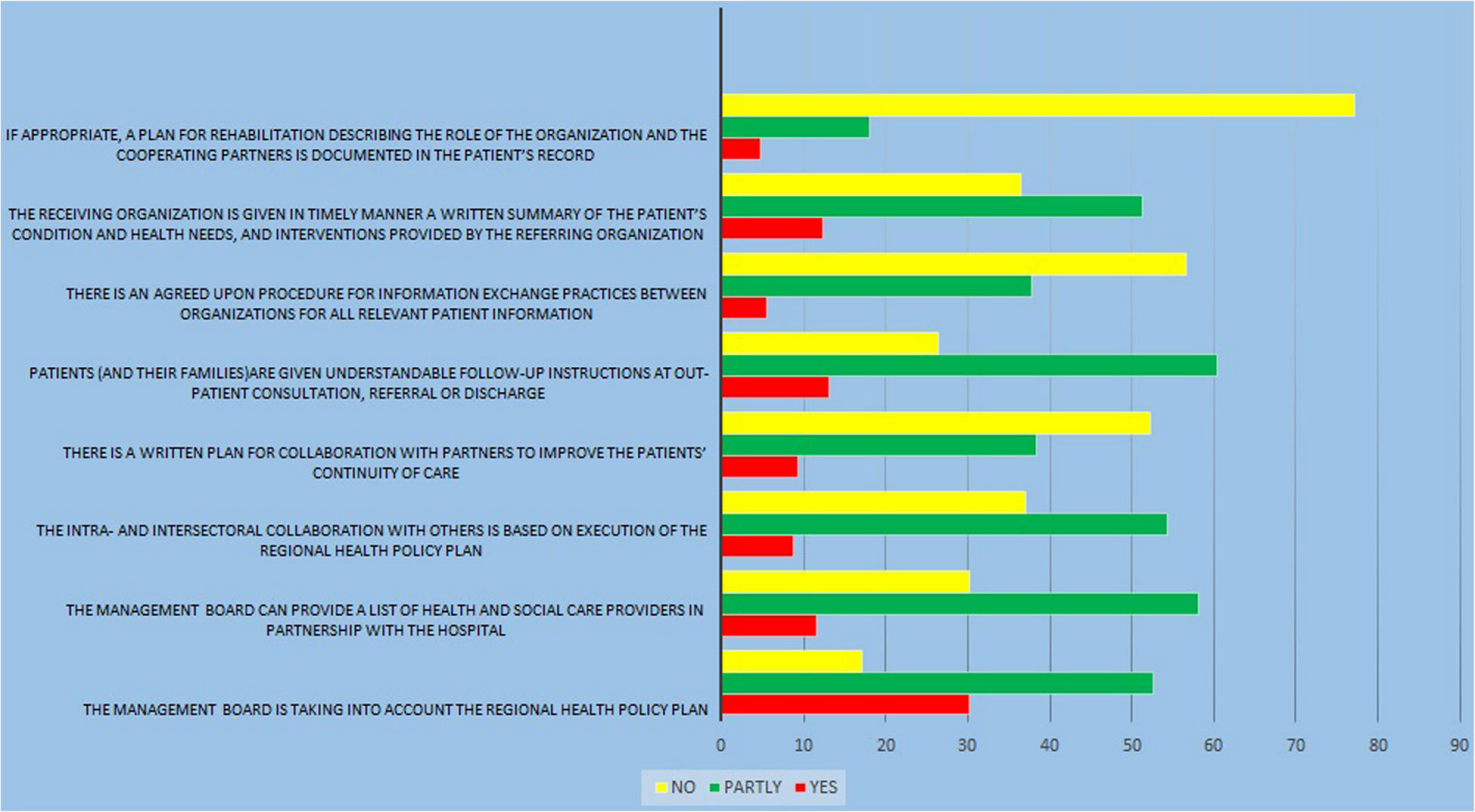
Standard5; Continuity and cooperation

The last dimension (Fig. 5) of the HP deals with the planned approach of the organization in cooperation with other health service provider organizations [11]. The results on this dimension showed that cooperation exists to some degree and the managers and health decision makers consider it. But there were some barriers such as the lack of a plan for rehabilitation and collaboration of other organizations or non-government organizations (NGOs), lack of agreement between the organization and patients’ information for communication and information exchange.

## Discussion

The aim of this study was to assess the standards of HPHs at Shahid Madani Hospital in Tabriz, Iran, to identify the levels, barriers and facilitators of these standards. The finding showed a correlation between the HPH activities and the organizational post and working experience of the personnel. The possible cause of the correlation might be that by increasing the working experience of the personnel, they acquire more education and experience in HP standards and cooperation with other organizations. Also, working in different posts might lead to increased professional abilities.

The HPH activities in the studied hospital obtain an overall moderate score which is consistent with the findings of Afshari et al. in 9 hospitals of Isfahan, Iran. They reported that the HP activities in the studied hospitals were at the intermediate level and suggested that the policy makers and the managers be involved in the HP programs to integrate the policies and to implement the guidelines [24].

In the present study the lowest scores of HPH activities in this hospital belong to the management policy and the patient assessment standards which have effects on the other standards too. There were also barriers such as lack of specific programs and budgets for promoting health, and lack of guidelines for alcohol and smoking as well as reassessment of patients’ needs at discharge, in these two standards. Given that these problems are more relevant to the organization’s management and policy, for the organization’s managers to upgrade the standard of HPHs, budgets and specific plans need to be set for these standards.

Study of Johnson and Baum stated that the primary concept of the HPH in the mind of the participants was implementing the fifth strategy of the Ottawa Charter which is re-orienting the healthcare services towards prevention of illness and promotion of health. While using the hospitals as an environment for HP means that the other strategies of the Ottawa charter should be practiced including: building healthy public policy, creating supporting environments, strengthening community action, developing personal skills [19].

In this study, the average score of the patient assessment, building a healthy and safe environment and Continuity and cooperation were 1.45, 1.63 and 1.53 respectively. Also, the results showed that in these standards, there were some barriers such as lack of patient satisfaction assessment programs, plans to assess the health issues of employees, as well as training new staff and the lack of agreement on all patient information. Thus, the hospital should focus on all standards of the HPH, specially the two mentioned dimensions.

Tountas et al. stated that planning for HP should consider factors like support and acceptance of the hospital to institute HP principles regarding the viewpoints and benefits of various involved personnel, availability of reliable documents of the patients, and providing activities related to health problems of the patients and personnel [25]. The authorities of this hospital can use the barriers and facilitators of the standards of HPHs identified in this study. The hospital can improve health promotion standards by establishing specific programs on HPHs [26], special funding and training experienced people in the field of health promotion activities [27], encouraging cooperation between the other health service providers and NGOs, measuring the satisfaction of the patients, making the policies transparent, involving the personnel and the patients [27–30].

Also, considering these factors in planning and promoting health at the center, it can reduce costs, mortality and disabilities, as well as increase the level of satisfaction of visitors to the center [2, 30, 31]. Yaghoubi et al. when developing a conceptual model for hospitals committed to the HP, also stated that before developing the HP policies, they must have a good knowledge about the surrounding society, such as community’s epidemiological situatio, an appropriate assessment to differentiate the needs of the culture conflicts and the values ​​of different groups of people in the community. They need to design a system for assessing and developing Health promotion. Also, they have to assess social and health Issues and the pattern of diseases in the community. So that, the programs and health promotion projects based on community needs would be implement [32].

However, there are also a number of limitations to the study including: the assessment of HPHs standards only in a public health center. Participants in this study were selected on a convenience basis and the lack of motivation among personnel such as low knowledge about HPH and HP interventions, the lack of key individuals’ participation as well.

## Conclusion

Since the most important dimension of HP standards is the Management policy, which affects the other dimensions too, the authorities of the studied hospital should be more involved in the HP activities. Considering the fact that of the nearly 1000 hospitals of the country, only five hospitals are member of the International Network of Health Promoting Hospitals and Health Services, and as the results of the studies show the almost all Iranian hospitals suffer from the same problems in this regard, the findings of this study can be applied in other hospitals too.

Finally, it is imperative that health managers and policy makers in the country create health promotion policies and budgets for hospitals with the aim of joining HPH networks, and train specialists in this field to improve the infrastructure of health promotion hospitals, and create programs for educating people to participate in these programs, and encourage them to participate and, through appropriate methods, monitor and reform these programs.

## Abbreviations

COPD: Chronic Obstructive Pulmonary Disease
HP: Health Promotion
HPH: Health Promoting Hospital
IUMS: Iran University of Medical Sciences
NCDs: Non-Communicable Diseases
NGOs: Non-Government Organizations
WHO: World Health Organization
